# Chitosan-based nano-scaffolds as antileishmanial wound dressing in BALB/c mice treatment: Characterization and design of tissue regeneration

**DOI:** 10.22038/ijbms.2020.41361.9770

**Published:** 2020-06

**Authors:** Seyyed Javad Seyyed Tabaei, Mohsen Rahimi, Mohsen Akbaribazm, Seyed Ali Ziai, Minoo Sadri, Seyed Reza Shahrokhi, Mitra Sadat Rezaei

**Affiliations:** 1 Department of Parasitology and Mycology, School of Medicine, Shahid Beheshti University of Medical Sciences, Tehran, Iran; 2 Department of Parasitology and Mycology, School of Medicine, Student Research Committee, Shahid Beheshti University of Medical Sciences, Tehran, Iran; 3 Anatomical Sciences, Fertility and Infertility Research Center, Kermanshah University of Medical Sciences, Kermanshah, Iran; 4 Department of Pharmacology, School of Medicine, Shahid Beheshti University of Medical Sciences, Tehran, Iran; 5 Department of Biochemistry and Biophysics, Education and Research Center of Science and Biotechnology, Malek Ashtar University of Technology, Tehran, Iran; 6 Doctor of Veterinary Medicine (D.V.M.), Rasht, Iran; 7 Virology Research Center, National Research Institute of Tuberculosis and Lung Diseases (NRITLD), Shahid Beheshti University of Medical Sciences, Tehran, Iran; 8 Pathology Department, School of Medicine, Shahid Beheshti University of Medical Sciences, Tehran, Iran

**Keywords:** Bandages, Berberine, Drug-delivery system, Nanostructures, Wound healing

## Abstract

**Objective(s):**

Rapid healing of cutaneous leishmaniasis as one of the most important parasitic diseases leads to the decrease of scars and prevention of a great threat to the looks of the affected people. Today, the use of nano-scaffolds is rapidly increasing in tissue engineering and regenerative medicine with structures similar to the target tissue. Chitosan (CS) is a bioactive polymer with antimicrobial and accelerating features of healing wounds, which is commonly used in biomedicine. This study aimed to investigate the effects of CS/polyethylene oxide (PEO)/berberine (BBR) nanofibers on the experimental ulcers of *Leishmania* *major* in BALB/c mice.

**Materials and Methods:**

CS/PEO/BBR nanofibers were prepared by the electrospinning method, and their morphology was examined by SEM, TEM, and AFM. Then, water absorption, stability, biocompatibility, porosity, and drug release from nano-scaffolds were explored. Afterward, 28 BALB/c mice infected with the parasite were randomly divided into control and experimental groups, and their wounds were dressed with the produced nano-scaffolds. Finally, the effect of nanobandage on the animals was investigated by macroscopic, histopathologic, and *in vivo* imaging examinations.

**Results:**

The prepared nanofibers were completely uniform, cylindrical, bead-free, and biocompatible with an average diameter of 94±12 nm and had appropriate drug release. In addition, the reduced skin ulcer diameter (*P=*0.000), parasite burden (*P=*0.003), changes in the epidermis (*P=*0.023), and dermis (*P=*0.032) indicated significantly strong effectiveness of the produced nano-scaffolds against leishmania ulcers.

**Conclusion:**

Studies showed that CS/PEO/BBR nanofibers have a positive effect on the rapid healing of leishmania ulcers. Future studies should focus on other chronic ulcers treatment.

## Introduction

One of the main challenges faced by physicians is to find a method to improve the recovery process and regeneration of the damaged tissues. Numerous factors, such as large fractures, traumas, burns, accidents, and infectious factors can lead to ulcers, including cutaneous leishmaniasis. Leishmaniasis has been observed in 98 countries of the world, and more than 350 million people are threatened by this disease. The disease appears as three types of cutaneous, mucocutaneous, and visceral. The cutaneous form is more common worldwide and accounts for more than two-thirds of the annual incidence (1, 2). With regard to the several side effects of the common drugs for treating leishmaniasis, it is necessary to find new treatments with fewer side effects on other tissues (3).

Today, nanobiotechnology has led to significant changes due to its use in various scientific fields. Therefore, development of natural drugs has been significantly considered because of the lowest side effects. Tissue engineering is one of these fields in restoration and regeneration of damaged tissues. While constructing a tissue, it is necessary to have a scaffold with a suitable physical and mechanical structure so that it can provide the ground for migration, growth, and cellular proliferation in order to achieve a specific morphology, adhesion, creation of new tissue, and replacing it with the damaged tissue in the body (4-7).

Wound healing consists of hemostasis, inflammation, proliferation, and remodeling. After preventing hemorrhage in the hemostasis phase, infiltration of neutrophils and macrophages is observed for preventing infections in the inflammatory phase. Inflammatory cells secrete several mediators needed for granulation of tissue formation. Transforming growth factor-β (TGF-β) is an inflammatory cytokine that regulates the rate of cell proliferation and apoptosis. Then, numerous growth factors such as vascular endothelial growth factor (VEGF) and fibroblast growth factor (FGF) are secreted at the site of the ulcer. All pro-angiogenic agents initiate to proliferate and differentiate cells; however, excessive inflammation, by itself, reduces the amount of healing by producing reactive oxygen species (ROS) and proteases. Then, the proliferative phase begins via proliferation of fibroblasts and secretion of collagen in order to form an extracellular matrix. During regeneration, fibers of the mature collagen and wound heal by forming a scar. The capillary formation results from the proliferation of endothelial cells, which provide oxygen and nutrients to fibroblasts. The deeper the wound, the more scars will be created. When the recovery phase stops at any of the above phases, the wound will be chronic. Cotton gauze is traditionally used to dress the wound. Today, modern dressings include interactive and bioactive; interactive dressings include films, sponges, hydrogels, and so forth, which have antibacterial effects, while bioactive dressings interfere actively with the wound healing (8, 9).

Production of electrospun nanofibers can meet important regeneration needs, such as the ability to penetrate gases (e.g., oxygen), protect the wound against secondary infection, and prevent dehydration. An ideal dressing creates a higher porosity and can provide a more suitable barrier against microorganisms. Moreover, it should absorb wound secretions, prevent infection of the wound surface, provide proper conditions for the gas exchange of the wound, and can be easily removed from the tissue without any damages. Wound dressing with electrospun nanofibers has major advantages than the conventional dressings, because electrospun nanofibers, due to their very high surface and microscopic structure, begin to produce necessary signals to absorb fibroblasts into the dermal layer, which accelerate regenerating the damaged tissue by speeding up the formation of extracellular matrix (7).

Natural polymers such as chitosan (CS) are widely used in biomedicine due to acceptable properties of biocompatible, antimicrobial, non-toxic, giant cell migration, fibroblast activation, stimulation of type IV collagen synthesis, and increased healing rate of ulcers. CS is a hydrophilic polymer, but it needs another hydrophilic polymer, such as polyethylene oxide (PEO) due to its high molecular weight and high viscosity in order to produce nanofibers. PEO is placed in the CS chains and prevents repulsion of similar charges and disruption of its chains. This compound increases the CS electrospinning potential, leads to an increase in the hydrophilic property of the nano-scaffold and better wound dressing with the aim of protecting, inhibiting invasion of microorganisms, and accelerating the regeneration process (6).

As the main and natural ingredient of *Berberis vulgaris*, berberine (BBR), an isoquinoline alkaloid, has anti-inflammatory effects by inhibiting the activation of 5’ adenosine monophosphate-activated protein kinase (AMPK), nuclear factor kappa light chain enhancer of activated B cells (NF-κB) and activator protein 1 (AP-1) pathways and related inflammatory factors such as IL-6, IL-8, IL-13, TNF-α and IFN-γ*,* which has been approved in several studies, and possesses a high potential for eliminating various parasites and *Leishmania* strains by regulating the mitogen activated protein kinase (MAPK) pathway in macrophages. However, researchers are still investigating this ingredient (4, 5).

With regard to the effective role of the polymer nanofibers in wound healing and drug transfer, this study aimed to produce a composite nano-wound dressing of CS/PEO/BBR nanofibers and determine its effect on the regeneration of cutaneous leishmaniasis in BALB/c mice.

## Materials and Methods

***Chemicals and instruments***

Medium molecular weight CS, PEO with 900 KD MW, Berberine chloride hydrate (BBR), and MTT powder [3-(4.5-dimethylthiazol-2-yl)-2.5-diphenyl tetrazolium bromide)] were purchased from Sigma-Aldrich (USA). *Leishmania major *^GFP^ (MRHO/IR/75/ER) was obtained from Department of Immunology, School of Medicine, Shahid Beheshti University of Medical Sciences, Tehran, Iran.

Electrospinning instrument (Full Option Lab ES, NANOAZMA), scanning electron microscope (SEM) (AIS 2100, Seron Technology), transmission electron microscope (TEM) (EM 900, ZIESS), atomic force microscope (AFM) (NanoWizard-II, JPK instruments), FT-IR device (Nexus 670, Thermo Nicolet), UV-Vis spectrometer (SPECORD 210, Analytik Jena AG) and *In vivo* Imaging System F Pro (Kodak) were used. 

***Preparation of CS/PEO polymeric solution (90:10)***

Because of insolubility of CS alone, first, it was required to add 0.04 g of PEO to 0.24 g of CS in acetic acid 0.5 (M). With minor modification of a previously described method, 0.5 wt% of Triton X-100 and 10 wt% of dimethyl sulfoxide (DMSO) were added to this solution as emulsifier and co-solvent, respectively. Then, the prepared solutions were mixed using a magnetic stirrer at 500 rpm for 24 hr at 37 ^°^C to yield a homogeneous solution (10). 

***Preparation of CS/PEO/BBR solution***

**Figure 1:**
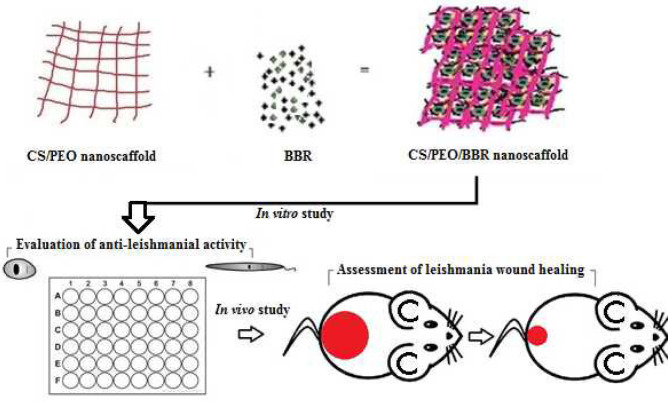
Schematic representation of scaffold preparation steps and evaluation of the antileishmanial activity of prepared nanobandage under *in vitro* and *in vivo* conditions

In this stage, BBR with percentages of 0.5–20 wt% was added to polymeric solutions of CS/PEO at weight ratio of 90:10 and stirred well for 5 hr at 37 ^°^C to produce a clear and transparent solution prior to electrospinning. Then, the solution was poured into a 2 mL syringe using a 0.5 mm needle stick, and the nanofibers were prepared using the electrospinning technique (feed rate: 0.1 ml/hr, voltage: 15-16 kV, tip-to-target distance: 12-14 cm, and rotating speed: 300 rpm)(Figure 1).

***Characterization of electrospun CS/PEO/BBR nanofibers***

The surface of obtained nanofibers was sprayed with gold (Au) and their morphology was observed by an SEM microscope with different magnifications. For TEM image preparation, 200 mesh grids were used. (10, 11).

***Evaluation of nanofiber stability and water absorption***

To simulate skin conditions, the temperature and pH of phosphate*-*buffered saline (PBS) were changed to 37 ^°^C and 5.5, respectively. Then, 25 mg of obtained nanofibers were weighed and immersed in the mentioned solution for one day. Afterward, morphological structure changes of the nanofiber mats were investigated using SEM.

The water absorption of prepared samples was calculated by using the [(W_s_−W_0_) / W_0_]×100 formula, where W_0_=Initial weight of the dry sample and W_s_=Swollen weight of the sample at equilibrium. (12)

***Fourier transform infrared (FT-IR ) spectroscopy***

The samples were mixed in 1:10 ratio with potassium bromide to prepare particles smaller than 2 μm and were compacted to a 0.25 mm tablet. Afterward, the spectra were reported by an FT-IR device (10, 11).

***Porosity (ɛ) measurement***

First of all, nano-scaffolds were immersed in a cylinder containing 96% ethanol (V_1_) for one day. The total volume of the solution with nanofibers and the volume difference (V_1_-V_2_) were reported as V_2_ and V_3_, respectively. The porosity was then calculated according to the [(V_1_-V_3_)/(V_2_-V_3_)]×100 formula (13).

***Cross-linking of nanofibers***

Hydrophilic polymers are dissolved in a medium containing water similar to the body and take a gelatinous form. One of the important methods for preparing water-soluble polymer nanofibers is to establish a cross-link between hydrophilic polymers, which prevents destruction of nano-scaffolds under *in vivo* conditions.

For this, electrospun nanofibers were placed on top of the aqueous glutaraldehyde (25%) in a vacuum desiccator for one day at room conditions. To remove the unreacted glutaraldehyde, the nanofibers were dried for 24 hr (14).

***Drug releasing template from nanofibers***

BBR release through nano-scaffold was evaluated after drawing the calibration curve at 229 nm by UV-Vis spectrometry in accordance with the Beer-Lambert law. Afterward, the drug release rate was assessed for up to two weeks (12, 15).

***Experimental groups***

28 adult female BALB/c mice (aged 6-8 weeks) were provided by Pasteur Institute of Iran and stored in an animal house with a temperature of 23±1 ^°^C and humidity of 40-60% and 12-hr light/12-hr dark cycle. Mice were fed the standard laboratory animal pellets and drinking water. Then, 1×10^6^ *Leishmania major *^GFP^ was injected subcutaneously at the base of the BALB/c tail. After about 4 weeks nodules appeared, which gradually turned into wounds at the base of their tails. The mice were randomly divided into 7 equal groups (16).

1. Negative (healthy) control group (NC) (0.5 mL PBS injection around the base of the tail) once a day.

2. Positive control group 1 (PC-1) (treatment with spilling PBS on the wound, daily).

3. Positive control group 2 (PC-2) (treatment with spilling nanofiber solvent, daily).

4. Treatment group 1 (MA) (treatment with injection meglumine antimoniate around the wound until the edge fades, twice a week)

5. Treatment group 2 (CS/PEO) (using CS/PEO nanofibers as wound dressings, daily)

6. Treatment group 3 (CS/PEO/BBR) (using CS/PEO/BBR 20 wt% nanofibers as wound dressings, daily).

7. Treatment group 4 (BBR) (using BBR 20 wt% ointment, daily). 

 All experiments were performed at a specified time of day for 30 days.

***Histopathological examination***

*Tissue preparation and sectioning*

The area of the wounds and spleens of BALB/c mice was calculated using the ImageJ software. For following up the antileishmanial effects of nano-bandage production, mice were anesthetized with intraperitoneal injection of 100 μl diazepam (10 mg/2 ml) and *in vivo* imaging was performed. Ultimately, with conformity to the ethics of working with laboratory animals, mice were euthanized. Skin ulcers and spleens each were isolated and the specimens were separately transferred to formalin 10%. Paraffin blocks were prepared and stained with hemotoxylin-eosin on 5-micron sections. After completion of this stage, the samples were examined histopathologically. 

***Parasite density estimation***

The parasite density in the skin was calculated using Table 1 and Leishman body unit (LU) in the spleen specimens using the following formula: Leishman body unit (LU)=number of amastigotes/1,000 nucleated cells×weight of spleen or liver (g)(17) (Table 1).

***Histopathological examination with stereological methods***

**Figure 2:**
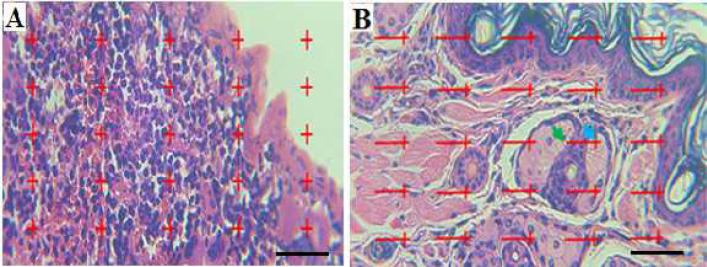
Point probe (25 points) for calculating the volumetric density of spleen. (A) The total number of points located on each structure is counted in each field of view and is compared with the total number of points in the volume measurement formula: Vv=∑P structure/∑P reference. Line probe (25 lines) to estimate the surface density of the skin structures. (B) The number of lines located on each structure (blue arrow)(∑p), the length of each of the lines in the probe is given by linear magnification (l/p) and the number of lines located on the internal part of structures (green arrow) (Σl). Finally, count the numbers in the following to calculate the density of the level: Sv=2× Σl/Σp × l/p (H&E staining, Scale bar: 100 μm, ×40)

The volume of spleens was determined by Cavallier’s method after estimating the tissue shrinkage rate. To this end, first, the primary volume of spleens was measured by immersion technique, and Isotropic Uniform Random (IUR) sections were prepared by the orientator method. The point probe method was used to calculate the relative volume of structures (volume of the red (splenic cords and sinuses)/white pulp, interstitial tissue, vessels, capsule, and trabecula) (Figure 2A). Finally, the relative volume of each structure was multiplied by the reference volume (after the fraction of the coefficient of shrinkage) to calculate the final volume of each structure (18).

To measure the mean thickness of different layers in the skin and its structures, the epidermal (corneal and cellular), dermal, and hypodermal thicknesses were measured using Nikon light microscopy equipped with a KEcam (KEcam Technologies, Lekki Lagos, Nigeria) and Top view software (version 3.7). The images were processed using Adobe Photoshop CC (Adobe system, San Jose, CA, USA) (Figure 2B) (19).

***Histopathological examination with pathological changes test***

In order to compare the rate of lymphocyte infiltration and tissue repair from a magnification of 100 and 400, based on the extent of lymphocytes spread, observation of scars, fibrous tissue formation, and tissue damage destruction in the field of vision were ranked from 0 to 4 in the following way:

1. 0-25% field of view has lymphocytic infiltration and there is no scar tissue or fibrous tissue, and tissue architectures are maintained (normal mode).

2. 25-50% of the field of vision has lymphatic infiltration and tissue architecture is destroyed.

3. 50-75% of the field of view has lymphocytic infiltration and the fibrous tissue with a surface less than 10% of the field of view is observed and the tissue architecture is disappearing.

4. 75-100% of the field of vision has lymphocytic infiltration, and the fibrosis level exceeds 30%, and in addition to the destruction of tissue architecture, scar also occurs (20).

***Statistical analysis***

IBM SPSS Statistics software package (version 24) was used for statistical analysis. Differences were determined using one-way ANOVA, followed by LSD comparison. One sample t-test and Tukey’s test were used for stereological parameter analysis and comparing each parameter between the groups. *P*<0.05 was considered significant.

## Results

***Evaluation of CS/PEO nanofibers (90:10) with and without BBR***

SEM images of CS/PEO nanofibers (90:10) indicated uniform, cylindrical, and bead-free nanofibers with an average diameter of 70±15 nm, which increased as the drug concentration increased (94±12 nm).

**Figure 3:**
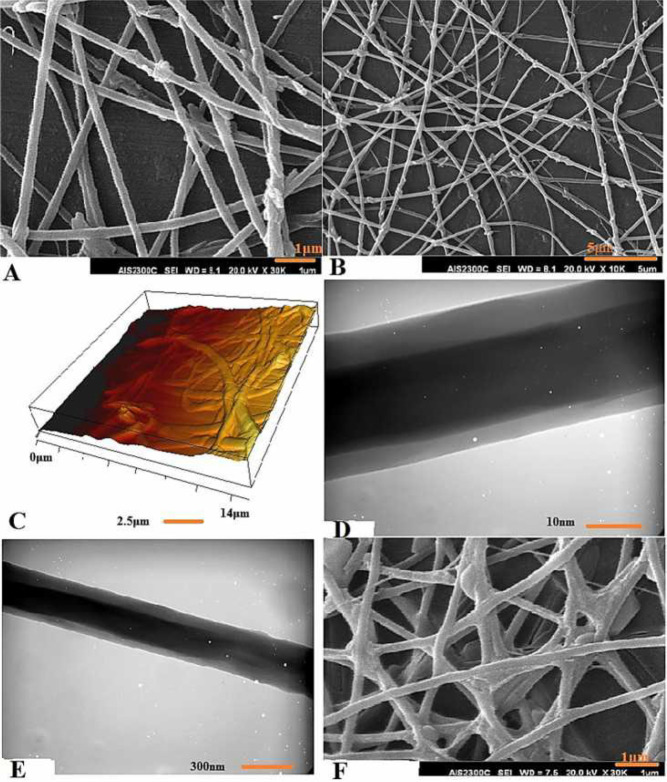
SEM images of electrospun CS/PEO 90:10 nanofibers containing BBR 20 wt% (A, B) in various magnifications; AFM image of a 3-D structure of prepared nanofibers after BBR 20 wt% loading (C); TEM images of electrospun CS/PEO/BBR 20 wt% nanofibers (D, E) in various magnifications; Stability study of cross-linked CS/PEO/BBR 20 wt% nanofibers after 24 hr floating in PBS at 37 ^°^C, pH 5.5 (F)

AFM and TEM images also demonstrated acceptable 3-D structure and suitable core-shell structure of the produced nanofibers (Figures 3A-E).

***Analysis of nanofiber stability and water absorption***

SEM images showed that the average diameter of the nanofibers increased by 191±22.6 nm after networking with glutaraldehyde under optimum conditions in the CS/PEO/BBR 20 wt% nanofibers and they maintained their structure in the aquatic environment (Figure 3F).

**Figure 4:**
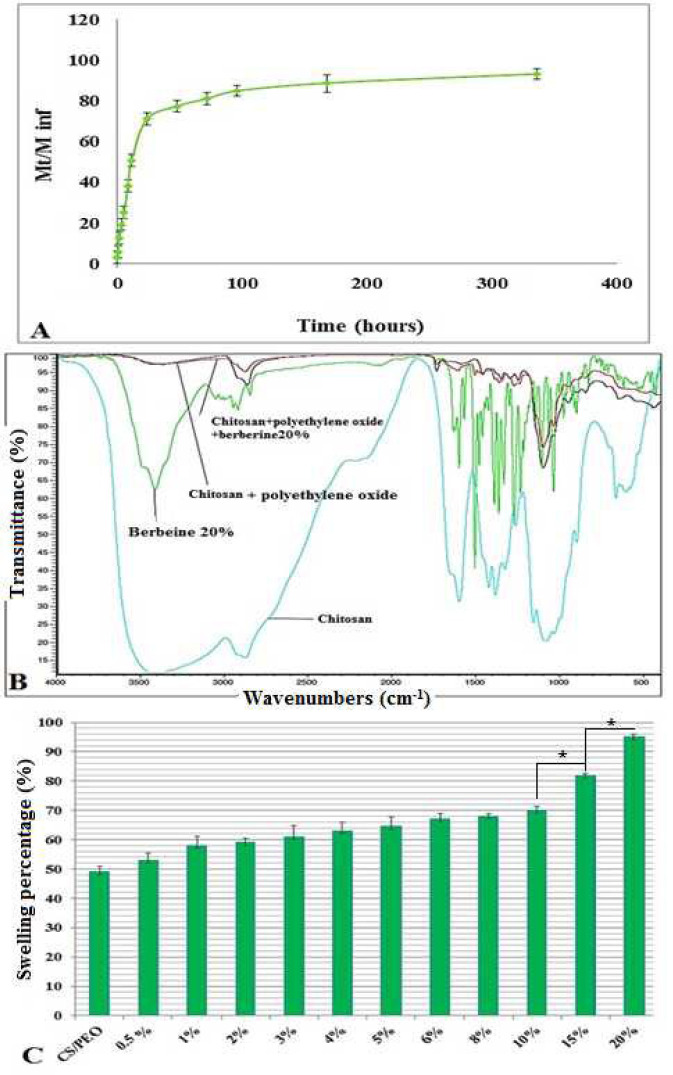
The cumulative BBR release from electrospun mats based on time (A); The overlay FT-IR spectrums of CS, BBR, and obtained nanofibers (B); The water absorption diagram of prepared nanofibers in PBS, 24 hr, pH 5.5 at 37 ^°^C (C)

The water absorption results showed an increase in nanofibers swelling by enhancing BBR concentrations. (Figure 4).

***FT-IR spectroscopy of nanofibers***

The FT-IR spectrum of electrospun nanofibers in a range from 400 to 4000 cm^-1^ at 24 ^°^C is shown in Figure 4. In the present study, functional groups in CS, BBR, and CS/PEO, CS/PEO/BBR 20 wt% nano-scaffold were investigated.

***Evaluation of BBR release from composite nanofibers***

The release rate of the drug from the scaffold was 50% after 12 hr, 70.7% on the first day, 77.1% on the second day, and 80.8% on the third day. Over time, the rate of drug release declined gradually, and the rate of BBR release from the scaffold continued for two weeks (94.98%)(Figure 4).

***Analysis of nanofibers porosity (ɛ)***

The porosity percentage analysis revealed that the obtained scaffolds had a porosity of about 90%. Achieving more than 80% porosity in tissue engineering is essential for the uniform distribution of cells throughout the scaffold.

***Antileishmanial effects of composite nanofibers on the parasite under in vivo conditions***

*Evaluation of the changes in weight and area of ​​the wound and spleen in BALB/c mice*

In NC, the size of the spleen was not increased, and no behavioral changes and mortality were observed until the end of the study.

In the PC-1 and 2, the average wound​​ and spleen areas showed a significant increase (*P*=0.003 and *P*=0.032, respectively) at the end of treatment.

In both groups, swelling and wounds in the hands and feet, broken tails, and wound prevalence in the back of the bodies of mice were observed from the beginning to the end of treatment.

Average wound ​​area in MA had a significant decrease compared with PC-1 and 2 groups (*P*=0.000 and *P*=0.001, respectively) at the end of treatment compared with the beginning of the treatment. When meglumine was injected, the area of ​​the spleen was significantly decreased (*P*=0.024) compared with the PC groups.

Average wound area in CS/PEO decreased compared with PC-1 and 2 (*P*=0.000), and significant increase (*P*=0.003) was found compared with MA. The area of ​​the spleen in this group was significantly higher than those of the PC groups (*P*=0.019).

The average wound ​​area had a significant decrease in CS/PEO/BBR compared with PC groups (*P*=0.000). The spleen area reduced significantly compared with PC-1 (*P*=0.021) and CS/PEO groups (*P*=0.000).

Although the spleen area experienced an apparent decrease in the average area compared with PC-2 (*P*=0.183) and MA (*P*=0.938) and had an apparent increase in the average area compared with the BBR group (*P*=0.487), there was no significant mean difference between the groups.

The average wound ​​area in BBR represented a significant decrease compared with PC groups after the end of treatment (*P*=0.000).

**Figure 5:**
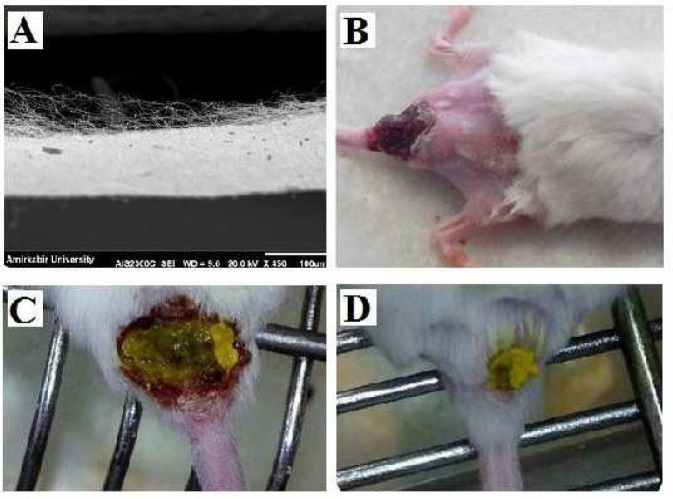
Cross-sectional SEM image of obtained nanobandage (A); Shaved dorsal back of infected mouse before *in vivo* imaging process (B); Images of using nanobandage on *Leishmania major* ulcer in BALB/c mouse at Onset (C) and End (D) of the treatment

Moreover, the average spleen ​​area had a statistically significant reduction compared with PC-1 (*P*=0.004), PC-2 (*P*=0.050), and CS/PEO groups (*P*=0.000) (Figure 5 and Table 2).

***Histopathology and in vivo imaging follow-up***

**Figure 6:**
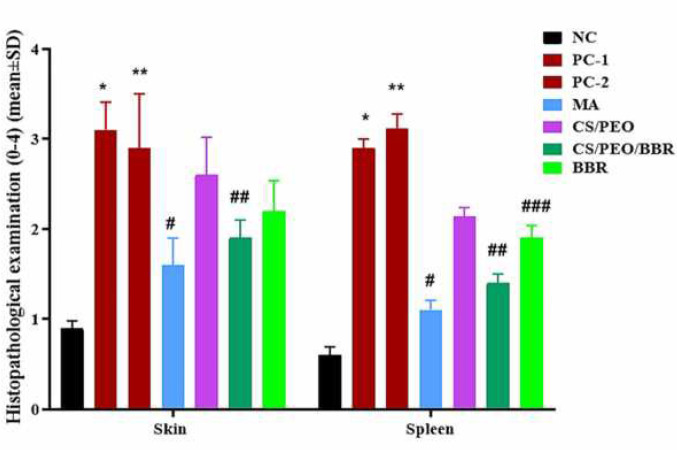
Histopathological examination in negative (healthy) control group (NC), positive control group 1 (PC-1), positive control group 2 (PC-2), treatment group 1 (MA, meglumine antimoniate), treatment group 2 (CS/PEO), treatment group 3 (CS/PEO/BBR), and treatment group 4 (BBR) mean±SD. Based on the amount of lymphocyte infiltration, tissue fibrosis, congestion, repair and preservation of tissue architecture have been evaluated

^*^ (*P<*0.05) statistically significant compared positive control group 1 (PC-1) and ^**^ positive control group 2 (PC-2) with negative control (NC). ^#^ (*P<*0.05) statistically significant compared positive control groups 1 and 2 (PC-1 and 2) with treatment group 1 (MA), ^##^ (*P<*0.05) with treatment group 3 (CS/PEO) and ^###^ (*P<*0.05) with treatment group 4 (BBR)

Macroscopic images of the wound in the experimental groups after the treatment confirm the wound healing process acceleration in the groups treated with CS/PEO/BBR compared with PC-1 and 2 groups. Also, wound healing in PC-2 was not significantly different from that of PC-1. In microscopic examination, the keratinized layer, epidermal layers, dermal layers, hypoderm, hair follicles, sebaceous glands, and blood vessels were detectable (Figure 6).

After comparing the results of histopathologic findings in the spleen, lesions and histopathological parameters significantly increased in PC-1 (*P*=0.006) and 2 (*P*=0.008). Meanwhile, these parameters and lesions were significantly reduced in the treatment groups with MA (*P*=0.001), CS/PEO/BBR (*P*=0.011), and BBR (*P*=0.038). Similar results were observed in the histopathologic findings in the skin of the experimental groups in PC-1 (*P*=0.001) and 2 (*P*=0.006) compared with the NC group, while these parameters and lesions were significantly reduced in the treatment groups with MA (*P*=0.011) and CS/PEO/BBR (*P*=0.042). The remaining treatment groups also showed a decrease in histopathological lesions, but this reduction was not statistically significant. 

**Figure 7:**
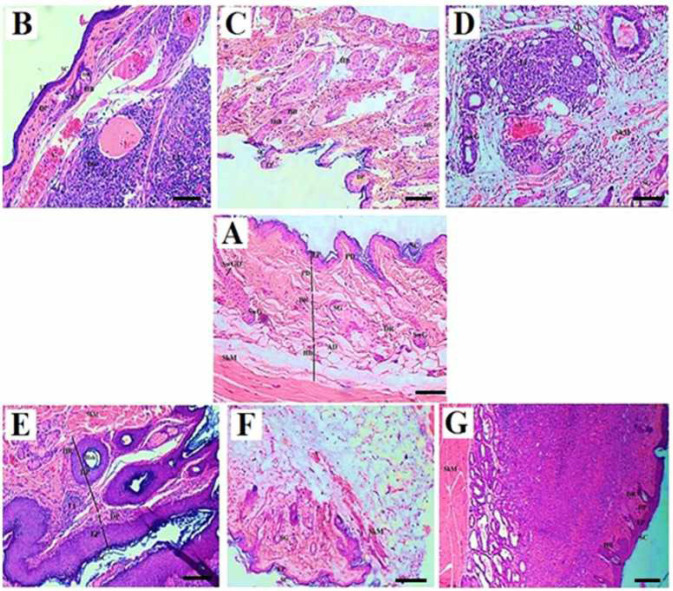
Histopathological images of *Leishmania major* skin lesions; Negative control, NC (A); Positive controls, PC 1 and 2 (B, C); Meglumine antimoniate group, MA (D); Chitosan/polyethylene oxide nanobandage group, CS/PEO (E); Chitosan/polyethylene oxide/berberine 20 wt% nanobandage group, CS/PEO/BBR (F); Berberine 20 wt% ointment, BBR (G).

**Figure 8.**
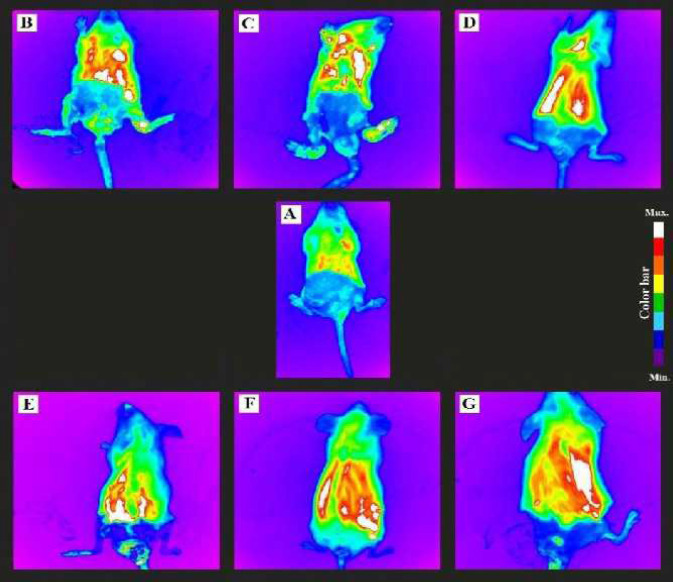
*In vivo* images of *Leishmania major* wound healing; Negative control, NC (A); Positive controls, PC 1 and 2 (B, C); Meglumine antimoniate group, MA (D); Chitosan/polyethylene oxide nanobandage group, CS/PEO (E); Chitosan/polyethylene oxide/berberine 20 wt% nanobandage group, CS/PEO/BBR (F); Berberine 20 wt% ointment, BBR (G)

In PC-1 and 2, an invasive injured epidermal epithelium was observed that continued until the hypoderm, and a large part of the tissue had a large necrosis, the glands of the sebaceous had disappeared, the keratinous layer was not seen, the hair follicles were destroyed, and the fibroblast and collagen cells were not observed. Some necrotic vessels and infiltration of inflammatory cells with large scrapes, as well as a large number of histiocytic cells combined with 6+ parasitic score were observed (Figures 7B, C). An *in vivo* imaging study confirmed the severe infection of the wound by the parasite and its release to the inguinal lymph nodes (Figures 8B, C). 

In MA, there is very little necrosis. A large number of histocytes are observed but the tissue is free of parasites (Figure 7D). The germs of sebaceous are lost but the wound bed of some fibroblastic cells and also *in vivo* imaging indicate parasite removal and decreased size of the ulcer (Figure 8D).

In CS/PEO, the epidermis is full of inflammatory cells. Sebaceous glands have disappeared and hair follicles have sometimes been degraded and fibroblasts are rarely seen. A large number of histocyte cells were observed with a parasitic score of 3+ (Figure 7E). Tracing with *in vivo *imaging also showed a decrease in the presence of parasites in the wound (Figure 8E).

In CS/PEO/BBR, necrosis is much lower compared with CS/PEO group and a large number of histocytes are seen. The number of parasites has decreased (1+). The number of inflammatory cells has dropped a lot (Figure 7F). As *in* *vivo* imaging illustrates, the number of parasites has been severely reduced and accelerated healing process (worsening wound closure) is well observed (Figure 8F).

In BBR, a large number of inflammatory and histocyte cells are present with parasitic infection with score 3+ (Figure 7G). Tracking with *in vivo* imaging also showed a decrease in the presence of parasites in the wound (Figure 8G) (Table 3).

**Figure 9:**
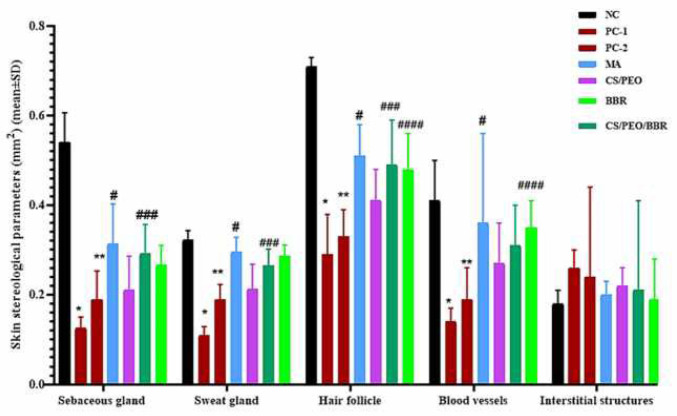
The density of the sebaceous and sweat gland, hair follicle, blood vessels, and interstitial structures in skin (mm2) according to surface density (SV) and reference volume in negative (healthy) control group (NC), positive control group 1 (PC-1), positive control group 2 (PC-2), treatment group 1 (MA, meglumine antimoniate), treatment group 2 (CS/PEO), treatment group 3 (CS/ PEO/BBR), and treatment group 4 (BBR) mean±SD

^*^ (*P<*0.05) statistically significant compared with positive control group 1 (PC-1) and ^**^ positive control group 2 (PC-2) with negative control (NC). ^#^ (*P<*0.05) statistically significant compared with positive control group (PC) 1 and 2 with treatment group 1 (MA), ## (*P<*0.05) with treatment group 2 (CS/PEO), ^###^ (*P<*0.05) with treatment group 3 (CS/PEO/BBR) and ^####^ (*P<*0.05) with treatment group 4 (BBR)

In the results of the stereological findings related to skin structures, the area (surface density) of the sebaceous and sweat glands were significantly decreased in PC-1 (*P*=0.004) and 2 (*P*=0.009) compared with NC. However, the area of sebaceous glands in MA (*P*=0.009) and CS/PEO/BBR (*P*=0.033), and sweat glands in MA (*P*=0.021) and CS/PEO/BBR (*P*=0.040) has been significantly increased. In examining the area occupied by other structures in the skin, the area occupied by hair follicles decreased significantly in PC-1 (*P=*0.004) and 2 (*P*=0.009), while in MA (*P*=0.011), CS/PEO/BBR (*P*=0.038), and BBR (*P*=0.042) it significantly increased. The area occupied by blood vessels in PC-1 (*P*=0.008) and 2 (*P*=0.013) was lower than that in NC, while in MA (*P*=0.024) and BBR it increased (*P*=0.031). With regard to the area occupied by interstitial tissue, despite its increase in PC-1 and 2, there was a significant difference between the groups in comparison with NC, and its relative reduction in the treatment groups compared with PC groups was not observed (Figure 9).

**Figure 10:**
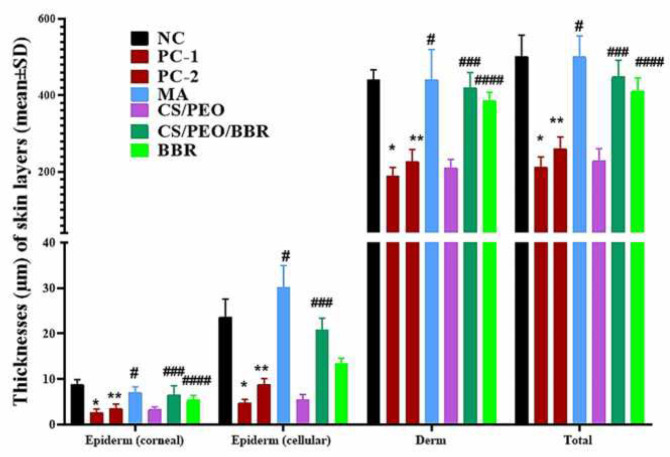
Thicknesses (μm) of skin layers in negative (healthy) control group (NC), positive control group 1 (PC-1), positive control group 2 (PC-2), treatment group 1 (MA, meglumine antimoniate), treatment group 2 (CS/PEO), treatment group 3 (CS/PEO/BBR), and treatment group 4 (BBR) mean±SD

^*^ (*P<*0.05) statistically significant compared with positive control group 1 (PC-1) and ^**^ positive control group 2 (PC-2) with negative control (NC). ^#^ (*P<*0.05) statistically significant compared with positive control group (PC) 1 and 2 with treatment group 1 (MA), ^##^ (*P<*0.05) with treatment group 2 (CS/PEO), ^###^ (*P<*0.05) with treatment group 3 (CS/PEO/BBR) and ^####^ (*P<*0.05) with treatment group 4 (BBR)

The results of skin stereological findings show the thickness of the horny (stratum corneum) and cellular layers of the epidermis significantly decreased in PC-1 (*P*=0.009) and 2 (*P*=0.018) compared with NC findings showed that the thickness of these layers was significantly higher in MA (*P*=0.006), CS/PEO/BBR (*P*=0.023) and BBR (*P*=0.041) increased. However, similar results were observed in the histopathologic findings of the skin of the treatment groups, while the dermal thickness in PC-1 (*P*=0.011) and 2 (*P*=0.033) were significantly higher compared with NC, and the thickness of these layers was significantly increased in MA (*P*=0.004), CS/PEO/BBR (*P*=0.016), and BBR (*P*=0.032) (Figure 10).

**Figure 11:**
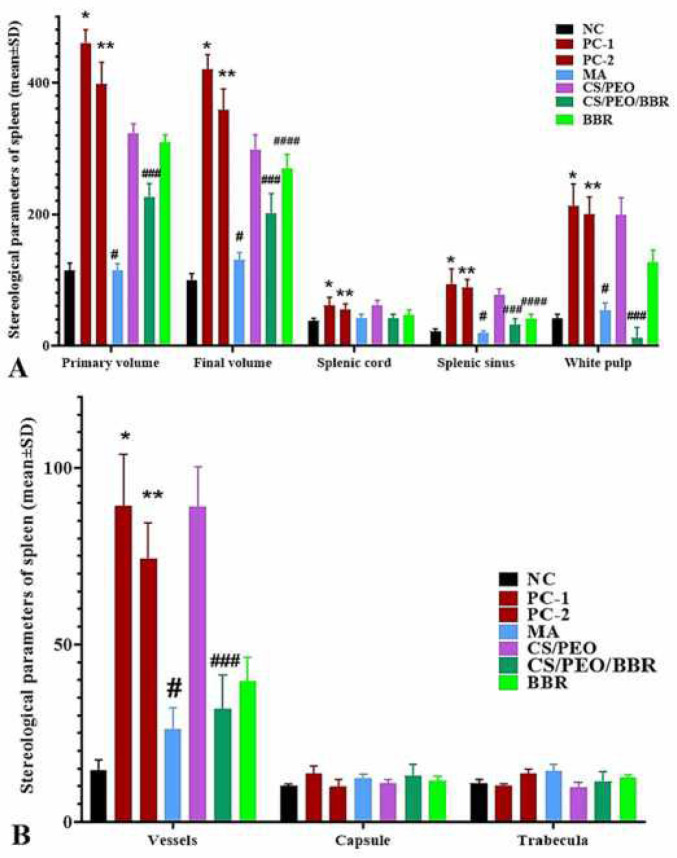
Primary and final volumes of spleen, red pulp (cord and sinus), white pulp (A) and vessels, capsule and trabecula (mm3) (B) in negative (healthy) control group (NC), positive control group 1 (PC-1), positive control group 2 (PC-2), treatment group 1 (MA, meglumine antimoniate), treatment group 2 (CS/PEO), treatment group 3 (CS/PEO/BBR), and treatment group 4 (BBR) mean±SD

^*^ (*P<*0.05) statistically significant compared with positive control group 1 (PC-1) and ^**^ positive control group 2 (PC-2) with negative control (NC). ^#^ (*P<*0.05) statistically significant compared with positive control group (PC) 1 and 2 with treatment group 1 (MA), ## (P<0.05) with treatment group 2 (CS/PEO), ^###^ (*P<*0.05) with treatment group 3 (CS/PEO/BBR) and ^####^ (*P<*0.05) with treatment group 4 (BBR)

**Figure 12:**
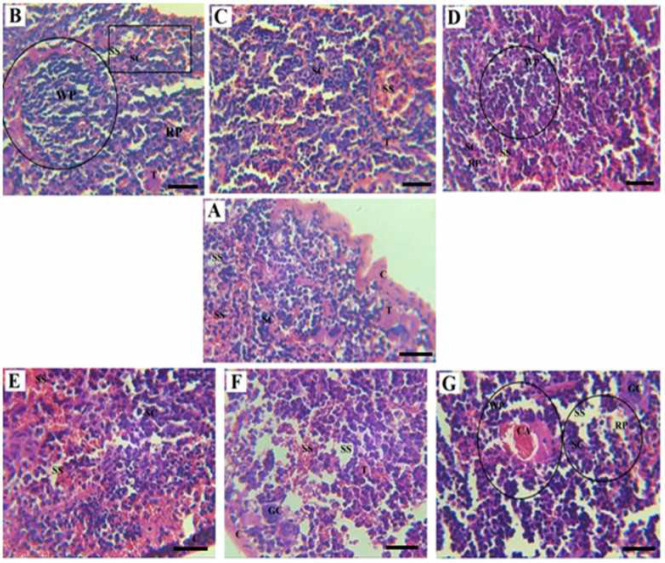
Histopathological images of *Leishmania major* infection in the spleen; Negative control, NC (A); Positive controls, PC 1 and 2 (B, C); Meglumine antimoniate group, MA (D); Chitosan/polyethylene oxide nanobandage group, CS/PEO (E); Chitosan/polyethylene oxide/berberine 20 wt% nanobandage group, CS/PEO/BBR (F); Berberine 20 wt% ointment, BBR (G)

After comparing the results of the volume of structures in the spleen, the final volume (*P*=0.008), sinusoids (*P*=0.012), white pulp (*P*=0.006) and vessels (*P*=0.009) in the NC compared with PC-1 and 2 increased significantly. The MA had a significant decrease in the final volume (*P*=0.004), primary volume (*P*=0.011), sinusoids (*P*=0.001), white pulp (*P*=0.018) and vessels (*P*=0.001) compared with PC-1 and 2 (*P*=0.021) and white pulp (*P*=0.018) PC-1 and 2. CS/PEO/BBR had a significant decrease in the final volume (*P*=0.017), primary volume (*P*=0.023), vessels (*P*= 0.014), sinusoids (*P*=0.011) and white pulp (*P*=0.009) compared with PC-1 and 2. BBR also showed a significant decrease in the final volume of the spleen (*P*=0.029) and sinusoids (*P*=0.031) compared with PC-1 and 2 (Figures 11 and 12).

## Discussion

This study indicated that the use of CS/PEO nanofibers containing BBR on the leishmanial ulcers of the BALB/c mice led to rapid wound healing in terms of macroscopic and microscopic aspects, and also significantly declined the wound diameter. 

A previous study on BBR shows this material has an antileishmanial effect by adjusting the pathway of mitogen-activated protein kinase (MAPK) in macrophages (4). Another study on the therapeutic potential of isoquinoline alkaloids present in several herbal plants confirmed the anti-inflammatory effects of BBR in *in vitro* environment, as well as on the animals (5). The results of a study indicated that BBR plays an inhibitory effect on *Leishmania *in dogs (21). The present study, however, has proven strongly the antileishmanial effects of BBR.

The results of comparison of meglumine injection with topical application of paromomycin and methyl benzethonium chloride in the treatment of leishmania cutaneous lesions indicated higher effect of topical drugs that were consistent with the results of our study (22).

The results of the examination of chitin hydrogel and CS as wound dressing indicated recurrent epithelialization of the wound treated with chitin hydrogel and replacement of granulated tissues with fibrosis and hair follicles after 7 days. In this study, chitin hydrogel was considered an effective factor in wound healing due to its easy use and high efficacy. In our study, in addition to wound healing and formation of hair follicles, angiogenesis was also observed as an important option in the regeneration process (23). One of the studies used a wound dressing consisting of two layers (upper layer as carboxymethyl chitin hydrogel and lower layer as an antimicrobial agent) as the wound dressing. The hydrogel layer acted as a mechanical barrier, absorbed wound secretions up to 4 times more than its weight, and prevented accumulation of fluids in the wound and infection through microbial inhibition. Moreover, the nano-scaffold produced in the present study had a high absorbability, such that it kept the wound completely dry, and could eliminate the parasite (24).

Researchers conducted a study to prevent bacterial infection using beta-chitin based wound dressing containing silver sulfuridiazine, and indicated that the produced dressing (bandage) had an acceptable permeability to oxygen, and maintained its antibacterial ability against *P.aeruginosa* for seven days. Moreover, histological examinations confirmed the proliferation of fibroblasts in the wound bed and reduction of infection. Our studies showed that drug release continues for 14 days. It should be noted that higher proliferation of fibroblasts was observed as in the above study (25).

Another study used CS as a wound dressing to examine faster wound healing rates. Results indicated that CS significantly accelerated the closure and healing of wounds in comparison with the control group. Histological examination also demonstrated a relatively complete tissue regeneration on days 2 to 4 in the wound treated with CS, which is in line with our study in terms of accelerated wound healing in the groups treated with CS nano-scaffolds (26).

Non-toxicity is the most important property of CS. Researchers used CS membranes to treat acute burn wounds and showed their ability as the wound dressing due to the potential of the produced bandage to control the moisture evaporation rates from wounds. In the present study, nanofibers obtained with a high porosity also contributed about 90% to the wound gas exchange (e.g., oxygen), which was in line with this study (27). 

The use of water-soluble chitin to increase the rate of wound healing on the rabbit ear showed that the produced ointment significantly increases the rate of regeneration and wound healing. The epithelium created in the study was significantly larger compared with the control group, and density of fibroblasts was higher in the wound under epithelium compared with chitin ointment, whereas the number of inflammatory cells was significantly declined in comparison to the control group, which indicates stimulation of wound regeneration and prevention of excessive scar formation. Our study also clearly showed the angiogenesis process and growth of the keratinized layer and hair follicles, which had similar results with our study (28).

The previous study on the effect of chitin nanofibrils linked to CS glycolate as spray, gel, and gauze in wound healing represented high porosity and acceptable permeability to oxygen scaffold. The produced scaffold, due to the apparent similarity to the extracellular matrix, resulted in the stimulation of migration, cell proliferation, and adhesion, which corresponds to our study (29).

Examination of microbial cultures on the scaffold obtained by CS nanofibers and PVA showed its antimicrobial property against *Pseudomonas aeruginosa*. Moreover, the results of the culture of fibroblasts confirmed their highly suitable growth on the resulting nano-scaffold. Emergence of fibroblasts is one of the most important stages in the wound healing process, which appear at the wound site when tissue is damaged so that they can regenerate the damaged tissue. This finding is compatible with our results (30). One of the studies electrospun the quaternizad CS (QCS) and polyvinyl pyrrolidone, and used the resulting scaffold as an acceptable wound dressing. Since CS has ammonium functional groups and attacks the cytoplasmic membrane of fungi and bacteria, it has antifungal and antibacterial properties. In our study, secondary infection was not observed in the groups treated with nano-scaffold bandage due to the above-mentioned properties, absorption of secretions, and dry wound. Thus, suitable conditions were provided for the growth and proliferation of the regenerated cells, and a faster improvement was observed in forming skin layers and faster closure of the wound (31).

Previous studies on the use of CS/polyurethane and carboxyethyl-CS/poly (vinyl alcohol) as the wound dressing and examination of the culture of fibroblast (L_929_) cells on them showed the ability of above-mentioned nano-scaffolds to bind and proliferate these cells. The results indicated that they could be used as dressing for treating burns and chronic ulcers. The CS/PEO/BBR nano-scaffold in our study also increased the regeneration rate, which was accompanied by higher rate of fibroblast proliferation, migration of the regenerative cells, and higher adhesion in the body of the living being (32, 33). Several studies demonstrated the effect of CS monomers and oligomers by increasing the activities of inflammatory cells, including polymorphonuclear leukocytes, macrophages, and fibroblasts, which is caused by the presence of CS in the wound, resulting in faster regeneration. This result is consistent with our findings (34, 35).

The results of the examination of the cutaneous wound regeneration in mice indicated that CS hydrogel increased migration, adhesion, and cellular proliferation. The effect of this hydrogel on the wounded skin after the treatment period revealed that the treatment group had acceptable regeneration, the epidermis was naturally formed, and keratinocytes appeared in their former shape. However, in the control group (without treatment), keratinocytes swelled, and epidermis was formed abnormally. Our study concluded that if regeneration is performed without dressing, it leads to a change in ​​the affected area. Therefore, addition of CS to the scaffold would improve healing and decrease the color change in the wound site (36).

Evaluation of CS gel with 1% silver sulfadiazine for treating the burn wound of mice indicated a clear increase in fibroblasts in the treated wounds compared with other groups, which is consistent with the results obtained in our study (37). The results of pathologic examinations on CS film and fucoidan in the healing of skin burn showed that fibroblasts appeared in the group treated with the above film earlier than other groups, and underwent a faster healing process than other groups (38). The previous studies’ results represented an increase in the number of fibroblasts through CS, which is related to its chemical properties and degree of deacetylation. Moreover, studying the effect of chitin and CS on the synthesis of collagen in wound healing demonstrated an increase in its production by CS. Examination of the regenerative effect of CS hydrogels as the wound dressing in burns indicated higher activity and greater number of inflammatory cells (38-40).

Moreover, an examination of the healing and antimicrobial effects of CS on the wound demonstrated that CS leads to stimulation, neutrophils, macrophages, and fibroblasts at different stages of wound healing. The results of this study showed infiltration of neutrophils at the early inflammatory phases, proliferation of fibroblasts, and production of collagen type III in the proliferation and maturation stages during the recovery process, which is compatible with our study results according to the higher number of fibroblasts and production of collagen (41, 42).

The scaffold should have suitable porosity, which is used for intracellular communication, cell migration, and blood vessel formation during the wound healing process. The porosity of the nano-scaffold produced in our study was calculated to be about 90%, which is consistent with the results of the previous studies (43, 44). 

The main objective of the present study was to accelerate the process of wound healing of leishmaniasis. Therefore, thickness of different skin layers and higher rates of angiogenesis were examined. Results indicated that the produced polysaccharide-based nano-scaffold could increase the rate of progression of epidermis from the healthy edge of the wound, and accelerate the wound healing of leishmaniasis.

The strength of the study is producing a natural nano-scaffold as a bioactive, biocompatible, biodegradable, non-toxic, non-allergen, and bio-absorbable wound dressing to regenerate wounds of *Leishmania major* by combining CS and BBR properties.

Since there is no information about the side effects of this dressing on humans, the lack of empirical examination of the dressing on humans is one of the limitations of the study.

**Table 1:** Calculating Leishman body density

**Average** ** parasite ** **density**	**Score**
> 100 parasites in each microscopic field*	6+
10-100 parasites in each microscopic field	5+
1-10 parasites in each microscopic field	4+
1-10 parasites in 10 microscopic fields	3+
1-10 parasites in 100 microscopic fields	2+
1-10 parasites in 1,000 microscopic fields	1+
0 parasite in 1,000 microscopic fields	0

* ×100

**Table 2:** Evaluation of skin wound healing changes and area measurement of spleen in negative (healthy) control group (NC), positive control group 1 (PC-1), positive control group 2 (PC-2), treatment group 1 (MA, meglumine antimoniate), treatment group 2 (CS/PEO), treatment group 3 (CS/PEO/BBR), treatment group 4 (BBR). Values are expressed as mean±SD

**Groups**	**Area measurement (mm** ^2^ **)**	
	**Skin wound healing changes**	**Spleen**
NC	-	129.937±26.746*
PC-1	53±17.568	204.80±26.345
PC-2	55.750±20.105	180.20±37.738
MA	-22.495±4.176*	151.80±34.50*
CS/PEO	2.540±0.751*	260.20±39.129*
CS/PEO/BBR	-26.450±6.534*	150.093±27.813*
BBR	-4.70±1.444*	134.625±19.11*

* Significant difference with PC-1 (one-way ANOVA)

Epidermis (EP), Dermis (D), Sebaceous gland (SGL), Stratum corneum (SC), Stratum basale (SB), Stratum granulosum (SG), Sweat gland (SwG), Excretory ducts of the sweat glands (SwGD), Dermis (Papillary) (DP), Dermis (Reticularis) (DR), Epidermal peg (Edp), Venule (V), Arteriol (A), Hair shaft (HSh), Inner root sheath (In), Outer root sheath (O), Capsule (C), Skeletal muscle (SkM), Adipose tissue (AD), Hypoderm (HD), Lymphocyte infiltration (LI) (H&E staining, Scale bar: 100 μm, ×40)

**Table 3:** Evaluation of parasite burden and mice weight changes in negative (healthy) control group (NC), positive control group 1 (PC-1), positive control group 2 (PC-2), treatment group 1 (MA, meglumine antimoniate), treatment group 2 (CS/PEO), treatment group 3 (CS/PEO/BBR), treatment group 4 (BBR). Values are expressed as mean±SD

**Groups**	**Parasite burden**		**Mice weight changes (%)**
	**Wound**	**Spleen**	
	** Score**	Leishman body unit	
NC	-	-	10.96 ±0.57
PC-1	6+	491±45.68	-2.27±0.25
PC-2	6+	505±48.566	-0.74±0.14*
MA	0	122±30.077*	-0.74±0.124*
CS/PEO	4+	459±45.04	5.18±0.866*
CS/PEO/BBR	1+	395±51.91*	11.93±0.947*
BBR	3+	433±41.88	1.32±0.26*

* Significant difference with PC-1 (one-way ANOVA)

Central arterioles (CA), White pulp (WP), Red pulp (RP), Splenic cord (the cords of Billroth) (SC), Splenic sinus (SS), Capsule of spleen (C), Trabecula of spleen (T) (H&E staining, Scale bar: 100 μm, ×40)

## Conclusion

This study produced a nanobandage for the first time that absorbs fluids and can provide the grounds for passing oxygen. It also keeps the wounds dry and protects the wounds against environmental contaminants. Examinations indicated proliferation of fibroblasts and endothelial cells, higher epithelialization, and rapid healing and regeneration of the damaged tissues. Therefore, the regeneration process was successful in the study, and the produced scaffold prevented deeper wounds and larger scars. Moreover, as the CS in the dressing is cationic, it contributes to this process by binding to anionic materials, polysaccharides, and proteins, and transferring the growth and drug factors.

Our results indicated higher ability of CS/PEO nanofibers dressing containing BBR in healing the cutaneous wounds caused by leishmaniasis in BALB/c mice with minimal scars, which can be considered a candidate for human treatment in future studies.
